# Soy Isoflavones in Nutritionally Relevant Amounts Have Varied Nutrigenomic Effects on Adipose Tissue

**DOI:** 10.3390/molecules20022310

**Published:** 2015-01-30

**Authors:** Elena Giordano, Alberto Dávalos, Maria Carmen Crespo, Joao Tomé-Carneiro, Diego Gómez-Coronado, Francesco Visioli

**Affiliations:** 1 Laboratory of Functional Foods, Madrid Institute for Advanced Studies (IMDEA)—Food, CEI UAM+CSIC, Carretera de Cantoblanco 8, Madrid 28049, Spain; E-Mails: elenagiord@gmail.com (E.G.); alberto.davalos@imdea.org (A.D.); carmen.crespo@imdea.org (M.C.C.); joao.estevao@imdea.org (J.T.-C.); 2 Servicio de Bioquímica-Investigación, Hospital Universitario Ramón y Cajal, Instituto Ramón y Cajal de Investigación Sanitaria (IRYCIS), Madrid 28034, Spain; E-Mail: diego.gomez@hrc.es; 3 CIBER de Fisiopatología de la Obesidad y Nutrición (CIBEROBN), Instituto de Salud Carlos III, Madrid 28029, Spain; 4 Department of Molecular Medicine, University of Padova, Padova 35121, Italy

**Keywords:** soy, isoflavones, leptin, genistein, daidzein

## Abstract

Soy consumption has been suggested to afford protection from cardiovascular disease (CVD). Indeed, accumulated albeit controversial evidence suggests that daily consumption of ≥25 g of soy protein with its associated phytochemicals intact can improve lipid profiles in hypercholesterolemic humans. However, the belief that soy foods and supplements positively impact human health has become increasingly controversial among the general public because of the reported estrogenic activities of soy isoflavones. In this study, we investigated the nutrigenomic actions of soy isoflavones (in nutritionally-relevant amounts) with a specific focus on the adipose tissue, due to its pivotal role in cardiometabolism. Young C57BL/6 mice were maintained for eight weeks under two different diet regimes: (1) purified control diet; or (2) purified control diet supplemented with 0.45 g% soybean dry purified extract (a genistein/daidzein mix). Soy isoflavones increased plasma total cholesterol concentrations and decreased triglyceride ones. Circulating leptin levels was also increased by soy consumption. Differentially expressed genes in adipose tissue were classified according to their role(s) in cellular or metabolic pathways. Our data show that soy isoflavones, administered in nutritionally-relevant amounts, have diverse nutrigenomic effects on adipose tissue. Taking into account the moderate average exposure to such molecules, their impact on cardiovascular health needs to be further investigated to resolve the issue of whether soy consumption does indeed increase or decrease cardiovascular risk.

## 1. Introduction

Soy consumption has been suggested to afford protection from cardiovascular disease (CVD) [1]. This notion largely stems from observation that CVD mortality rates are lower in Asian countries, where soy is an important part of the diet. Indeed, accumulated evidence suggests that daily consumption of ≥25 g of soy protein with its associated phytochemicals intact can improve lipid profiles in hypercholesterolemic humans, even though this has not been confirmed yet [2,3].

The purported cardioprotective effects of soy have been mostly attributed to its proteic constituents [4], yet soy also contains isoflavones, *i.e.*, phytoestrogens with potent estrogenic activity; notable examples include genistein, daidzein, and glycitein [5,6]. Numerous clinical studies claim benefits of genistein and daidzein in chemoprevention of breast and prostate cancer, cardiovascular disease, and osteoporosis as well as in relieving postmenopausal symptoms [7]. However, the belief that soy foods and supplements positively impact human health is becoming increasingly controversial among the lay public. This is due precisely to the estrogenic activities of soy isoflavones, which might negatively impact breast cancer risk, in particular among ER-positive breast cancer survivors [7].

In short, the effects of soy and its component on CVD and associated risk factors have not been fully elucidated. In this study, we investigated the nutrigenomic actions of soy isoflavones (in nutritionally-relevant amounts) with specific focus on the adipose tissue, due to its pivotal role in cardiometabolism.

## 2. Results and Discussion

### 2.1. Body Weight, Food Intake and Lipid Profiles

To investigate whether chronic feeding of soybean extract had an effect on body parameters, we measured body intake and food intake of C57BL/6 male mice maintained for eight weeks on either a purified control diet or control diet supplemented with 0.45% soybean extract, which is equivalent to 0.0661% of isoflavone mixture pure aglycones. No changes in such parameters were observed (Table 1A), but soy isoflavone supplementation induced a significant increase in plasma cholesterol concentrations (Table 1B) as compared with control diet. Conversely, triacylglycerol concentrations were significantly reduced.

**Table 1:** Body weight and food intake (**A**) and cholesterol and triglyceride circulating concentrations (**B**) in controls and in mice fed a 0.45% Soyselect^®^ diet.

**(A)**				
	**Body Weight**		**Food Intake**	
	g		g/day	
	**Control diet**	29.1 ± 2.1	**Control diet**	3.0 ± 0.1
	**0.45% Soyselect^®^**	27.7 ± 3.5	**0.45% Soyselect^®^**	2.7 ± 0.1
**(B)**				
	**Cholesterol**		**Triglycerides**	
	mg/dL plasma		mg/dL plasma	
	**Control diet**	117.5 ± 9.3	**Control diet**	67.6 ± 16.1
	**0.45% Soyselect^®^**	132.5 ± 2.9 *	**0.45% Soyselect^®^**	53.7 ± 8.14 *

Notes: Values are means ± SD; *n* = 7. Body weight, cholesterol, and triglyceride data are those obtained at the end of the study. Food intake data are those measured at the beginning and at the end of the study. * *p* < 0.05 as compared with controls.

### 2.2. Leptin Concentrations

**Figure 1:**
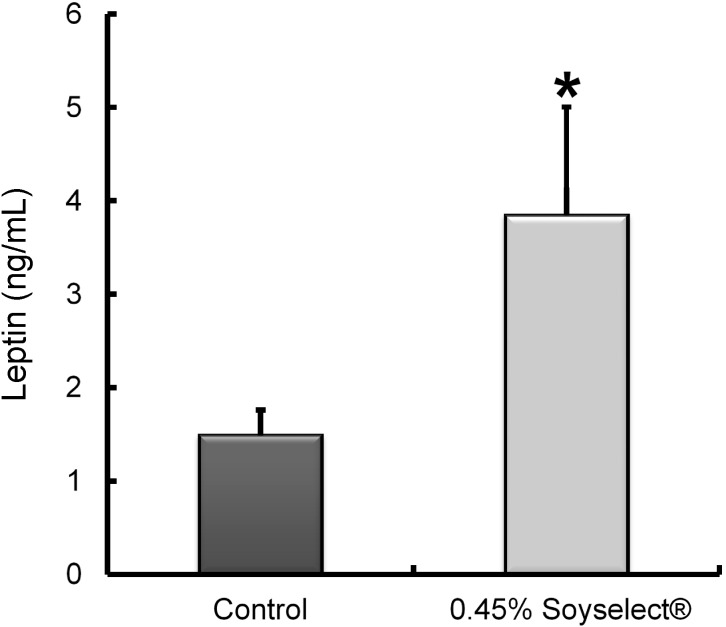
Leptin circulating concentrations in mice receiving either chow diet (control) or 0.45% Soyselect^®^ during eight weeks (*n* = 7 per group). * *p* < 0.05 as compared with controls.

Feeding 0.45% Soyselect^®^ led to a significant increase in circulating leptin concentrations which rose from 1.49 ± 0.27 to 3.85 ± 1.16 ng/mL (Figure 1).

### 2.3. Peritoneal Fat Genome Expression Profiles

Whole-transcriptome microarray analysis was carried out on perigonadal adipose tissue samples isolated from mice fed either purified control diet or 0.45% soybean extract diet. A total of 983 genes (437 downregulated and 546 upregulated) were differentially expressed at (a 2-fold cutoff with a q value of less than 5%). Complete lists of upregulated and downregulated genes are shown in Supplementary Tables S2 and S3, respectively.

**Figure 2:**
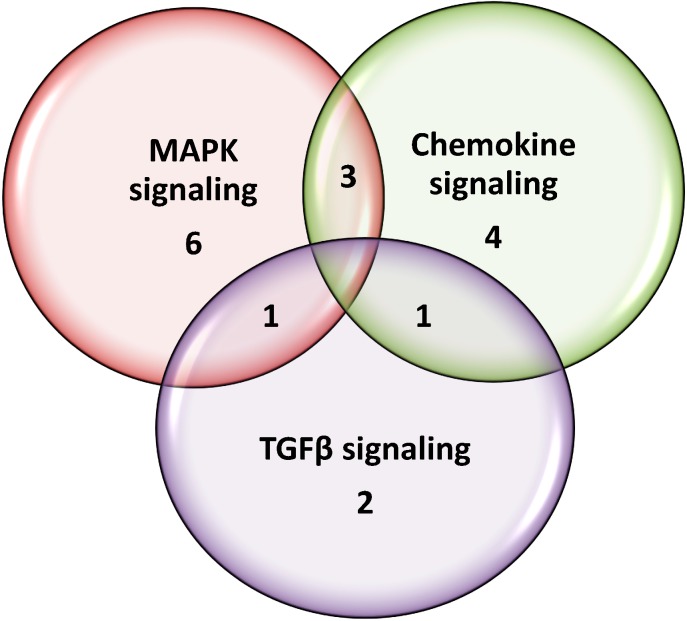
Venn diagram of selected pathway analyses. Pathway analyses of Soyselect^®^-modulated genes were performed and MAPK, TGFβ, and chemokine signaling were included as examples.

GENECODIS3 was used to perform biological pathway enrichment analyses of all downregulated genes in the interaction network (Table 2). The results show that a wide range of genes involved in many different biological pathways are modulated by Soyselect^®^. We selected three significantly enriched molecular functions, which include MAPK signaling, chemokine signaling, and TGFβ signaling, because they play pivotal roles in several cellular processes such as adipocyte differentiation and proliferation [8]. Specifically, a Venn diagram was constructed to evaluate the number of shared genes these three pathways: MAPK signaling [six genes], chemokine signaling [four genes], and TGFβ signaling [two genes] (Figure 2). The diagram shows that three genes, *i.e.*, MAPK1 (mitogen—activated protein kinase 1), PRKCB (protein kinase c, beta), and HRAS1 (Harvey rat sarcoma virus oncogene) are shared by the MAPK and chemokine groups; one gene, *i.e.*, MAPK1 is shared by all three groups and one gene, *i.e.*, ROCK1 (Rho-associated, coiled-coil containing protein kinase 1) is shared by chemokine and TGFβ signaling groups.

**Table 2:** Gene co-occurence annotations found by GeneCodis (molecular function) for genes downregulated by Soyselect^®^ in perigonadal white adipose tissue.

Genes	NGR	TNGR	NG	TNG	Hyp	Hyp *	Annotations
53 genes	1999	37681	53	365	3.21184 × 10^−11^	4.5287 × 10^−9^	GO:0000166: nucleotide binding (MF)
42 genes	1501	37681	42	365	9.08868 × 10^−10^	6.40752 × 10^−8^	GO:0016787: hydrolase activity (MF)
38 genes	1314	37681	38	365	2.51857 × 10^−9^	1.18373 × 10^−7^	GO:0005524: ATP binding (MF) GO:0000166: nucleotide binding (MF)
39 genes	1421	37681	39	365	6.44848 × 10^−9^	2.27309 × 10^−7^	GO:0005524: ATP binding (MF)
61 genes	2999	37681	61	365	2.77158 × 10^−8^	7.81585 × 10^−7^	GO:0005515: protein binding (MF)

*p*-values have been obtained through hypergeometric analysis (Hyp) corrected by FDR method (Hyp *) NGR, number of annotated genes in the reference list; NG, number of annotated genes in the input list.

### 2.4. Gene Expression Validation by qRT-PCR

**Figure 3:**
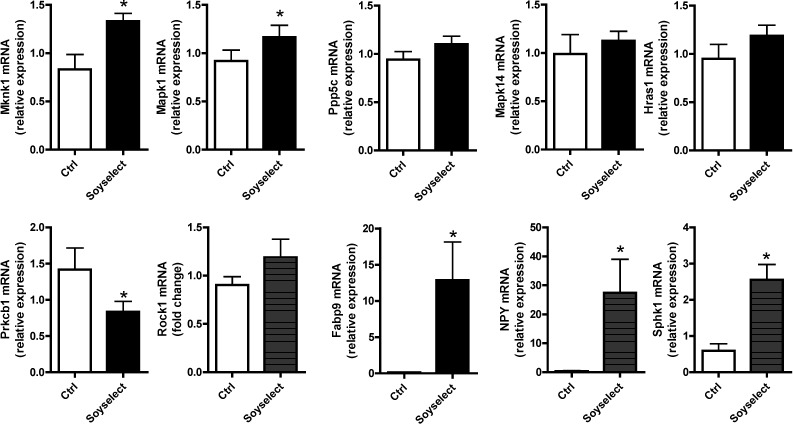
RT-qPCR validation of selected genes related to MAPK, TGFβ, chemokine signaling, and others related to lipid metabolism. Mice received either chow diet (ctrl) or 0.45% Soyselect^®^ during eight weeks. mRNA is given as relative expression (*n* = 6 per group). * *p* < 0.05 as compared with controls.

MAPK, TGFβ and chemokine signaling selected genes were analyzed by qRT-PCR (Figure 3) for validation. We also report other known genes related to lipid metabolism in adipose tissue and other highly modulated genes that came out from microarrays analysis, using a customized RT^2^ profiler PCR array (Supplementary Table S4).

Only Mknk1, Mapk1, and Prkcb1 were found to be modulated by the genistein/daidzein mix, while the other genes were not confirmed by qRT-PCR, suggesting either a modest effect of Soyselect^®^ on adipose tissue gene expression or lack of sensitivity of microarray experiments, which highlight the need for validation using qRT-PCR. However, other genes related to adipose biology and lipid metabolism were found to be modulated by Soyselect^®^, including the fatty acid binding protein 9 (Fabp9), neuropeptide Y (Npy), and sphingosine kinase 1 (Sphk1). In most cases, validated genes exhibited a more modest effect in their expression when evaluated by qRT-PCR than by microarrays.

### 2.5. Discussion

We have performed the first nutrigenomic study of soy isoflavones in adipose tissue. Our data show that these molecules, administered in nutritionally-relevant amounts [6,7,9], have diverse albeit modest effects on adipose tissue, which is an organ prominently involved in cardiometabolism [4]. As mentioned, the low cardiovascular mortality of some Asian countries, particularly Japan, has often been attributed to the high consumption of soy products, e.g., miso, natto, shoyu, soy beans, *etc.* [3,5]. Inadequate epidemiological research prevents from drawing firm conclusions [10] and it is noteworthy that, while most of the cardioprotective effects of soy have been ascribed to its proteic fraction [4,11,12], these products only contribute small amounts of protein to the whole diet. Therefore, the real contribution of soy protein to cardioprotection has been questioned [2]. In addition to putatively healthful proteins, soy is rich in isoflavones such as genistein and daidzein. These compounds are the subject of active research and current recommendations on their consumption are under intense scrutiny. In fact, isoflavones resemble 17-β-estradiol in structure, and as such are able to bind the estrogen receptor (ER) *in vitro*, behaving much as a natural selective estrogen receptor modulator (SERM) [13]. Therefore, soy isoflavones might act as tumor-promoting or tumor-inhibiting agents, likely depending on cell type, dose, and genetic predisposition. As far as the cardiovascular system is concerned, the current consensus appears to be that estrogens exert complicated and poorly understood effects on cardiovascular health [14,15]. When the effects of estrogens are analyzed for men and women separately, they clearly exert a protective effect on cardiovascular function during a woman’s childbearing years [14]. The challenge is now to convey these beneficial effects without unwanted steroid side effects on other organs and tissues, e.g., the breast [14]. Finally, genistein and daidzein have also been suggested to be neuroprotective and myorelaxant, namely in the detrusor muscle [16].

In adipose tissue, genistein and daidzein upregulate genes related to MAPK (Figure 2 and Figure 3) and downregulate chemokine signaling gene Prkcb1 (Figure 3). The MAPK family is attracting considerable attention because of its vast implications in signaling and crosstalk with other signaling networks [17]. Indeed, some authors are suggesting considering the possibility of targeting MAPK-mitochondria interactions in the prevention and treatment of heart disease [17]. Some polyphenols for which cardioprotective properties have been suggested—such as epigallocatechin gallate—do increase MAPK [18]. Yet, as recently reviewed by Hopkins [19], MAPK generally have strong prosurvival effects on macrophages and can have varied effects on scavenger receptor expression and foam cell formation. The resulting effects on atherosclerosis can be nuanced and difficult to predict [19]. Chemokines are expressed vessel wall cells and emigrated leukocytes. These molecules play important roles in atherosclerotic vascular disease, where they exert various functions including cell recruitment [20]. The actions of chemokines in vascular inflammation are stimulating research for therapeutic agents aimed at these molecules. In addition to the vascular district, chemokines play important roles in the infarcted heart, where unrestrained inflammation induces matrix degradation and cardiomyocyte apoptosis [21]. Consequently, inhibition of pro-inflammatory signals may be effective in patients with defective resolution of postinfarction inflammation. Of note, a recent publication reported that isoflavone supplementation induced anti-inflammatory gene expression in equol postmenopausal producers [22].

We also measured some circulating surrogate markers of cardiovascular disease. Notably, we recorded significantly increase circulating leptin concentrations in mice fed with soy isoflavones, namely Soyselect^®^ as compared with controls (Table 2). Even though the extent and precise nature of leptin’s contribution to cardiovascular disease is still unclear [23], accumulated evidence points to its negative role in vascular impairment and hypertension. It is worth noting that leptin can promote angiogenesis and induce neovascularization. In addition, high blood concentrations of leptin associated with obesity can lead to arterial endothelial dysfunctions, impaired arterial distensibility, and proliferation and migration of vascular smooth muscle cells. In humans, greater soy consumption appears to be associated with a lower presence of elevated total cholesterol, dyslipidemia, hyperuricemia and fewer cardiometabolic disturbances components [24]. No significant effect of soy isoflavones on blood pressure and endothelial molecules has been recorded [9,25]. A meta-analysis also reported that soy isoflavones have an effect of lowering blood pressure in hypertensive subjects, but not in normotensive subjects [26]. In short, the effects of soy isoflavones on the vasculature and blood pressure appear to be minimal in humans.

Soy isoflavones also increased total cholesterol while lowering triacylglycerol concentrations. The true human relevance of these findings is equivocal because, even though hypercholesterolemia is a known risk factor for CVD, lower triacylglycerols’ concentrations are also associated with better CV prognosis. It is noteworthy that cholesterol circulates in mice almost exclusively as high-density (HDL)-c [27]. As soy consumption brings about hypolipidemic effects [27], the true significance of the observed cholesterol increase is equivocal and deserves further investigation. We found that Fabp9, Npy, and Sphk1 were upregulated by Soyselect^®^ (Figure 3). Fabp9, Npy and Sphk1 are genes that regulate different aspects of lipid metabolism [28], adiposity [29], or adipocyte lipolysis [30]. Whether these and other Soyselect^®^-regulated genes are responsible for the observed effects on plasma cholesterol and triglycerides levels is not known and needs to be further investigated.

In conclusion, we add further evidence to the notion that soy isoflavones have assorted effects (both positive and negative) on cardiometabolic risk factors [31,32,33]. Keeping into account the moderate average exposure to such molecules, their impact on cardiovascular health need to be further investigated to solve the issue of whether soy consumption does indeed increase or decrease cardiovascular risk.

## 3. Experimental Section

### 3.1. Materials

A soybean purified extract containing isoflavones glycosides genistein and daidzein (14.7%; Soyselect^®^), was kindly donated by Indena (Milan, Italy). Soyselect^®^ is a standardized extract obtained from soy with a double standardization procedure and which contains 13%–17% of isoflavone glycosides genistein and daidzein and <18% of B-group saponins, as quantified by HPLC [34,35]. The product is prepared by extracting ripe whole soy beans or oil-free soy flour with aliphatic alcohols through an industrial manufacturing proprietary process [36,37]. One gram of extract also contains 0.058 g of protein, 0.035 g of fat, and 0.023 g of ash, with the remaining matter undefined (Supplementary Table S1). The batch (nr. 30432/M1) used in this study contained 14.7% isoflavone glycosides and 21.2% B-group saponins. Of note, saponins increase isoflavone’s bioavailability (unpublished data). SuperScript III First-Strand Synthesis System for RT-PCR was from Invitrogen (Madrid, Spain). Qiazol was from Qiagen (Izasa, Barcelona, Spain).

### 3.2. Animals and Diets

This investigation conforms to the Guide for the Care and Use of Laboratory Animals, published by the US National Research Council [38] and was approved by the Animal Experimentation Committee of the Universidad Complutense de Madrid.

Young C57BL/6 mice (2 months old, *n* = 14, *i.e.*, seven mice for each diet) were acclimatized in the animal facility on a 12:12 light/dark cycle, with the period of darkness between 7:00 a.m. and 7:00 p.m., for at least one week before experimentation. During this time, animals were fed a standard chow diet; food and water were given *ad libitum*. Then, mice were maintained for eight weeks under two different diet regimens (Research Diets, Inc. New Brunswick, NJ, USA): (1) purified control diet or (2) purified control diet supplemented with 0.45 g% soybean dry purified extract (genistein/daidzein mix). The final quantities of isoflavones mix in the diet correspond to ~0.661 mg/g of solid diet (0.0661%). Each diet provided 24.0%, 15.0%, and 61.0% kcalories from protein, fat, and carbohydrates, respectively. Their detailed composition is given in Table 3. To reduce diurnal variations, animals were sacrificed between 10:00 and 11:00 a.m., after an overnight fast. Mice were anesthetized with isoflurane and a midline incision was cut in the abdomen. Blood samples were collected from the vena cava. Heparin (0.4 mg/mL) was injected by means of the iliac vein and Hank’s balanced salt solution (HBSS; pH 7.4) was perfused through the portal vein for 2 min to remove blood. Tissues were quickly removed, washed twice in ice-cold HBSS, and snap-frozen and stored at −80 °C. In order to verify the dietary effects of soybean extract, body weight and food intake have been evaluated.

**Table 3:** Composition of the experimental diets.

	Control		Soyselect^®^	
	g% Kcal%		g% Kcal%	
Protein	23	24	23	24
Carbohydrate	60	61	60	61
Fat	6	15	6	15
**Ingredient**	**g/kg diet**			
Casein	244		244	
l-Cystein	3		3	
Corn Starch	318		318	
Maltodextrin 10	45		45	
Dextrose	250		250	
Cellulose	75		75	
Inulin	25		25	
Sunflower Oil	29.5		29.5	
Olive Oil	18.6		18.6	
Lard	18.5		18.5	
Mineral Mix S10026	10		10	
Dicalcium Phosphate	13		13	
Calcium Carbonate	5.5		16.5	
Potassium Citrate	5.5		16.5	
Vitamin Mix V10001	10		10	
Retinyl Acetate, 500,000 IU/g	0.048		0.048	
Choline Bitartrate	2		2	
Genistein/Daidzein mix	0		4.5	
Cholesterol	0.146		0.146	
Total	1083.84		1088.34	

### 3.3. Determination of Circulating Leptin Concentrations

Plasma concentrations of leptin were determined by ELISA kit, according to the manufacturer’s instructions (Mouse Leptin, 96-well plate assay, Millipore, Madrid, Spain).

### 3.4. Determination of Plasma Lipid Concentrations

Plasma cholesterol concentrations were determined by the Amplex Red cholesterol assay kit (Invitrogen, Madrid, Spain) following the manufacturer’s instructions. Plasma triglycerides were determined using an L-type triglyceride M test kit, according to the manufacturer’s instruction (Wako Chemicals, Neuss, Germany).

### 3.5. RNA Isolation and Analysis

Total RNA from perigonadal adipose tissue (100 mg) was isolated using Qiazol Lysis Reagent (Qiagen, Isaza, Barcelona, Spain) and QIAGEN RNeasy Mini kit columns (Qiagen). RNA was quantified using a NanoDrop-1000 Spectrophotometer (Thermo Fisher Scientific Inc., Madrid, Spain) and purity was assessed by measuring the ratio of absorbance at 260 nm and 280 nm. The quality of the RNA was tested in 1% formaldehyde-agarose gel stained with ethidium bromide (EtBr).

### 3.6. Microarray Hybridization

Gene expression profiles were assessed using Gene Expression Service with the Illumina MouseRef-8 v2 Expression BeadChip^®^ with Ambion Labelling. This BeadChip targets approximately 25,600 well-annotated RefSeq transcripts, over 19,100 unique genes.

Data were analyzed by using the GenomeStudio^TM^ Software (Illumina, San Diego, CA, USA) following the manufacturer’s instruction. Significant modulated genes were defined as those with an absolute fold change of >2.0 and an adjusted *p* value of <0.05.

Differentially expressed genes were classified according to their role(s) in cellular or metabolic pathways using the online GeneCodis analysis software for modular and singular enrichment analysis [39]. Gene Ontology (GO) analysis was also performed to describe the associated biological process of the differentially expressed genes overall [40].

### 3.7. Quantitative Real-Time PCR (qRT-PCR)

qRT-PCR of selected genes was performed to validate microarray results of adipose samples by using RT^2^ profiler PCR array (SABiosciences, Qiagen). The array was customized in 384 wells plates to contain a panel of genes specifically relevant to MAPK pathway, five different housekeeping genes and controls for genomic DNA contamination, reverse transcription and positive PCR controls. Dissociation curves were assessed to ensure the presence of a single amplicon. Reactions were performed with 50-fold diluted cDNA (1 ng/µL), 5 μL of USB VeryQuest SYBR Green qPCR Master Mix (2X) (Affymetrix, Madrid, Spain) and RNAse-free water being added to a final volume of 10 μL. Real-Time RT-PCR reactions were performed in 384-well plates and gene expression was determined using the 7900HT Real-Time PCR System (Life Technologies, Alcobendas, Spain). Cycling conditions were initial activation step at 95 °C for 15 min; 3-steps cycling for 40 cycle including denaturation at 94 °C for 15 s, annealing at 58 °C for 30 s and extension at 70 °C for 30 s; dissociation curve at 95 °C for 15 s, 60 °C for 15 s, 95 °C for 15 s. Gene expression was quantified using the ΔΔCt method and fold-change values were reported as 2^−(∆∆Ct)^.

### 3.8. Statistical Analysis

Statistical analyses were carried out using SPSS 19.0 (SPSS Inc., Chicago, IL, USA). Apart from gene expression, data independent samples t-test was used when the corresponding assumptions were met; otherwise the non-parametrical Wilcoxon Mann-Whitney test was employed. *p* < 0.05 was considered significant. Results are presented as means ± SD.

## Supplementary Materials

Supplementary materials can be accessed at: http://www.mdpi.com/1420-3049/20/02/2310/s1.
