# Supplementary material for: Soy Isoflavones in Nutritionally Relevant Amounts Have Varied Nutrigenomic Effects on Adipose Tissue
Source: Molecules. 2015 Jan 30;20(2):2310–22. doi: 10.3390/molecules20022310 (PMC6272387; doi:10.3390/molecules20022310)
Supplement: Supplementary file 1 [file molecules-20-02310-s001.pdf]

Supplementary Materials

Table S1. Chemical composition of the Soyselect batch used in this study.

**Energy Value**	58 (247)	kcal/100 g (kJ/100 g)
**Proteins (as sum of aminoacids after hydrolysis)**	1.93	g/100 g
**Total Fat**	1.4	g/100 g
• Saturated Fatty Acids	0.14	g/100 g
• Monounsaturated Fatty Acids	<0.05	g/100 g
• Polyunsaturated Fatty Acids	0.21	g/100 g
• trans-Fatty Acids	<0.05	g/100 g
**Total Dietary Fiber**	<0.5	g/100 g
**Sugars Composition**		
Fructose, anhydrous	2.10	g/100 g
Glucose, anhydrous	0.73	g/100 g
Lactose, anhydrous	<0.10	g/100 g
Saccharose (Sucrose), anhydrous	6.70	g/100 g
Maltose, anhydrous	<0.10	g/100 g
**Total Sugars**	9.53	g/100 g
**Moisture**	3.9	g/100 g
**Ash**	2.54	g/100 g
**Sodium**	37.0	mg/100 g
**Calcium**	2.82	mg/100 g
**Iron**	0.32	mg/100 g
**Cholesterol**	<0.10	mg/100 g
**Vitamin A** (retinol)	<0.01	mg/100 g
**B-Carotene**	<0.02	mg/100 g
**Vitamin C (Ascorbic Acid, HPLC)**	<2.5	mg/100 g

Table S2. Soy isoflavones mix induced genes.

DEFB37	DEFB21	EIF3K	PAPOLB	C730025P13RIK	BEX4
DEFB38	HEXB	PRPS1L1	ADAM5	STK22B	RASL2-9
PRM1	4921520G13RIK	ST6GALNAC2	1700021C14RIK	2700055A20RIK	LOC100044177
SPINT4	OTTMUSG00000016790	OXCT2B	LCN13	SQRDL	CYP4A12A
OTTMUSG00000016293	BHLHB2	BCAS2	MRPS18C	KPNB1	1190002A17RIK
DEFB23	NLRP14	GM128	KRT14	D430015B01RIK	1700093K21RIK
DEFB39	COX6C	SMARCD3	SEC61G	4921507P07RIK	SLC2A3
OAZ3	NTSR2	DEFB29	ODF4	CLK1	SLC30A6
SMCP	TCP11	D19ERTD721E	MAP1LC3B	TAX1BP1	BCKDHB
4931407G18RIK	HOXD4	1700025K23RIK	ODF3	CAGE1	CCNL1
NPY	COX8C	A330021E22RIK	SOCS7	HGFAC	IRGM
FABP9	ARL8A	1700010M22RIK	WDR20B	NPAL2	TWISTNB
9230002F21RIK	CRISP4	ACRV1	LOC100047749	1700010A17RIK	SLTM
SPAG11	CLDN11	AKTIP	GM1679	XLR4A	RAB5C
OTTMUSG00000015852	DDX25	STARD10	CCNO	BC048546	RNF133
SPINK2	DYNLT3	CCDC113	RASSF3	MRPL20	YWHAB
SPINK8	C130090K23RIK	ENPP2	CDH1	RPO1-1	1700067P10RIK
AY761185	CAMK2B	CCT6B	PRF1	1300007L22RIK	WBP4
TNP1	MYCBPAP	ZMAT1	1700026L06RIK	FRK	ZFP654
CUZD1	2410116G06RIK	PICALM	GRHL2	AGPAT3	ZBBX
SPINK11	SETX	COQ2	4921517D21RIK	RRN3	PABPC2
DEFB2	LOC100042777	GAPDHS	A530050D06RIK	CIZ1	PLP1
TSSK6	DDC8	KIF2B	ANKRD46	LOC100047200	RAD52
INDO	LOC677317	MNS1	SLC1A1	LOC100048020	EG385328
RPL3L	KCNC4	GSTT3	UBQLNL	4933402J07RIK	PLA2G4F
ADAM32	TCOF1	CPEB2	SPZ1	LIP1	DAPL1
WFDC15B	SLFNL1	LCN8	1700054O13RIK	HSP90B1	CFB
ODF1	KLHL10	1700020D05RIK	VDAC2	4922505E12RIK	1700001C02RIK
DEFB11	CCDC92	1700065I17RIK	4833420G17RIK	ERGIC2	TLE2

Table S2. *Cont.*

WFDC10	PRM3	SERF1	FYCO1	ELAVL1	2610101N10RIK
DEFB19	LRRC50	1700011H14RIK	ATP6V1A	UHRF1BP1L	1700019H03RIK
ALDH6A1	GRB7	4732415M23RIK	4933422H20RIK	RASGRF1	THAP4
SPINLW1	PTGDS	MOD1	EMG1	DYNLRB2	DEFB12
RSPH1	1810015C04RIK	CTNNB1	LLGL2	NDUFB9	IQCF4
BC051142	LOC100039532	1700029H14RIK	TMEM51	CGGBP1	TCTEX1D2
TCFAP2B	GSTT4	NOTCH1	ACTL7B	TRIM36	NIT1
LCN12	MFAP3L	4930412F15RIK	MED28	ARRDC2	1110002B05RIK
TULP2	LOC100048622	NT5C1B	TMCO5	ARHGEF4	1700026D08RIK
LDHC	HCFC1	CHCHD7	COL16A1	TNP2	HOXD3
BC030476	PTPRB	IDH2	CYB561	ADAM28	SETD1B
BC048679	GLDC	NXF1	HIST2H2BE	H3F3B	ISYNA1
PGK2	TUBA3B	BC038286	1700012L04RIK	KRT8	CC2D2A
LOC435023	SYTL4	CUL1	USP20	1700007K09RIK	M6PR
SPATA19	LYPD4	HADHB	SMAD1	ATP5A1	DEFB10
GSG1	SLC7A4	TMC5	4930526D03RIK	MBOAT1	ZPBP
PGAM2	DDIT4L	HILS1	PTGES3	ZDHHC4	OTUD5
ZNRF4	ATP13A4	1700094C09RIK	HSD17B10	HNRPAB	ZC3H15
UBQLN3	DEFB42	AQP9	1700057K13RIK	TBC1D8	2400003C14RIK
GYKL1	SPATA6	1700049L16RIK	TEKT1	BC020002	SPO11
SVS4	ACTL6A	PRDX6-RS1	CCT8	ATF4	CLIC3
SERPINA1F	LRRC57	BC049635	SMEK2	1110017D15RIK	SLC38A5
HDAC6	TDRD6	EG432867	4931406C07RIK	MAPBPIP	EG654465
CLGN	CST9	ENOPH1	EXOSC8	LRRC34	IL10RB
KCNK1	YBX2	ACTL7A	SKP1A	HUWE1	LOC100045098
TSGA8	BGLAP-RS1	BZW1	MDH1B	4930404H21RIK	5133400G04RIK
CLDN3	PFN3	EG433365	CUGBP2	GM614	SGPP1
LY6F	SMC5L1	CLCNKB	REC8	PAIP2	CAPZA3

Table S2. *Cont.*

DBIL5	P2RX2	NOL14	SATB1	HSD3B2	CYB5R1
SVS5	1700034E13RIK	ARID2	HOOK2	PURB	AKAP3
4922502D21RIK	AI314180	CLDN4	WDR23	CSTL1	MTMR12
EIF2S3Y	NUP210	LOC100048480	KCTD2	LRRC46	5830403L16RIK
TESK1	MLF1	ADCY8	OTTMUSG00000015859	GNPDA2	CRYBA4
PLS3	DEFB40	ZFP263	SNX1	NDFIP2	PRPS2
PDZK1	SPINK10	H2-AA	RPRM	GLB1	LAMA1
HOXB4	ESPN	TCTE3	STAG3	1700123L14RIK	PQLC1
LOC669168	CDH16	ODF2	DPEP3	TAF13	COX7A2
AP3B2	KRT18	C4BP	TRIP12	MGL1	DRBP1
RNASE9	SUSD4	LOC217341	GM1698	R3HDM2	4933405O20RIK
AKR1C19	SPACA3	SRP14	LYZL4	PBP2	PGM2
HBB-B1	ALDH1A1	EG665378	RGMA	CDC42EP3	TMEM176B
WFDC6B	GKAP1	1600016N20RIK	LOC100044779	1700018C11RIK	SLC44A4
ADAM3	ACTG2	DDX21	DDX3X	PRSS21	PTPRE
PACRG	DEFB30	1700080E11RIK	ACTR3	1700019D03RIK	SNF8
CYP17A1	ACSBG2	ISG20	ST13	ARL1	ANKRD10
SEC11C	HRASLS5	PGRMC1	HMGB4	4930451I11RIK	ZC3H6
NPC2	D11WSU47E	FHL5	AHCTF1	SPAG6	PIWIL1
DNAJC5B	TCP10A	CYP1B1	NDUFB10	ECSIT	CSPP1
DAZAP2	EMB	HSDL1	SPERT	PTPN1	IZUMO1
ACADSB	SELK	H1FNT	LYZL1	TTC29	D030013I16RIK
DEFB15	TESP1	QRICH2	PLAA	SERINC2	MGC107098
MRPL52	SVS7	TMED9	DCUN1D5	TECTA	RPS3A
RHBDL2	LTF	CRISP1	MARCH10	CCDC54	HSPA2
4930563D23RIK	HSPA1L	DNALI1	PRPS1	GALNTL5	AKAP4
WFDC13	S100A10	SPATA20	RAG1AP1	CATSPER3	1700030J22RIK

Table S2. *Cont.*

RBM35A	LOC100048703	OXCT2A	GOT2	DEFB25	AARD
LOC100047619	DEFB43	LCN5	ARMCX1	TXNDC8	HOXD8
POLR2G	ACTRT2	DEFB20	1700029J07RIK	TXNDC12	DEFB18
ZXDA	RNASE12	PROM2	TXNDC2	SLCO4A1	2900010M23RIK
YWHAZ	9230104L09RIK	LYZL6	PABPC1	TJP2	TRIM39
TUBA3A	DNAJC10	TEDDM1	4930503B20RIK	UPK1B	BC089491
ADAM7	LYAR	ENPP1	KCNH3	TPP2	SPHK1

Table S3. Soy isoflavones mix repressed genes.

LYZ2	C1QB	TMEM9	2410166I05RIK	THRSP	TAP1
NNAT	LAMC1	IGFBP6	AGTR1A	GTRGEO22	CRY2
GSN	ARSB	NID2	LOX	IL1RL1L	2010316F05RIK
APCDD1	ADRB3	DGAT2	HIST1H2BH	APOC3	ORC6L
SERPINA3C	2210021J22RIK	2010311D03RIK	REEP5	RGS16	HEPH
VIM	MFAP5	LOC100046393	LOC100044204	GEMIN7	C1QBP
TMEM45B	TMEM131	2310058J06RIK	DYNC1LI2	LEPREL2	EPB4.9
SSPN	GPX7	TMEM159	MRPL11	MAPK3	SH3KBP1
SVEP1	CIDEA	YIF1A	LOC654426	2600010E01RIK	ME2
AGPAT2	UGT1A10	MC2R	MCOLN1	NUMA1	BC031181
LUM	PTRF	CD248	CHCHD4	MRPS16	MCTS1
MAPK1	PI16	SLC19A1	MRPL3	LOC100045697	HLCS
HSPA8	PLEKHB2	HTATIP2	FRMD6	TSPAN7	PLD2
ATP5F1	LOC641240	CHPT1	SRPX	COL14A1	4631427C17RIK
GPR81	MKNK1	ZFP207	TNFRSF1A	PGAM1	MS4A6D
AKR1B7	APOA2	ACSM3	STX18	2610204L23RIK	MYCL1
G0S2	EMP3	GSTT1	IER3IP1	EG630499	MAEA

Table S3. *Cont.*

PXMP2	SPR	PPP2R1A	GBP2	VTN	RPS27A
CRTAP	TGFBR3	COX7A2L	GPX1	PCX	2510010F15RIK
H2-AB1	LPIN1	EMD	ATP6V0E2	TMEM100	ARHGDIB
HTRA1	MRPL9	H2-M3	MRPL53	CMTM7	MAPK1
GPR109A	SLC1A5	NAP1L1	PEG3	HTRA3	9030624J02RIK
FSTL1	ADH1	EAR4	MPP1	TCEAL8	LOC100048613
RARRES2	EMILIN2	LOC100047937	OSTF1	TGFBI	CHST12
COL15A1	ADFP	PRKCDBP	IFI35	CCDC80	TM6SF1
RNF4	GALM	BLCAP	CD83	RPL18	AU019823
CD14	MSN	IFITM2	GSTO1	RBPMS2	H3F3A
RNASET2	LOC100046650	LOC381629	HCLS1	1500032L24RIK	ZCCHC14
FFAR2	PSME2	GSTP2	OSTM1	SNRPB	CDKN2C
EG433923	NAPEPLD	GNB1	FKBP9	TRAPPC4	9330186A19RIK
SPARC	SNF1LK2	D430028G21RIK	FOXO1	JAZF1	MRPL4
ORM1	HP	LOC100048413	SRD5A3	PDDC1	A730042J05RIK
C1QC	GNG10	2010004A03RIK	SCP2	CRYAB	VKORC1
CXX1C	GFPT2	OXCT1	P2RX4	ANXA6	YKT6
H2AFZ	DHRS1	TMED10	G6PDX	ENTPD2	AMOTL2
ARL6IP5	FADS3	HNRPF	LOC100047012	LRG1	CPNE8
CYP4B1	LGALS3BP	PRKCB1	CSPG4	UQCRH	2700038C09RIK
SORBS1	MXRA8	ABCD2	SEPP1	SCOTIN	D830050J10RIK
TRF	HSD11B1	RAP2A	NID1	PRTN3	EMILIN1
NTRK2	ITGA7	FOLR2	COPS6	HIST1H2BF	1110031B06RIK
SAA3	IAH1	MOBK1B	GSTP1	CLPTM1	NOL5A
ACTN4	PHKG1	DOCK1	DPEP1	HRAS1	PER3
SYNGR2	NPR3	COQ9	1110002N22RIK	CD68	LDHA

Table S3. *Cont.*

S100A1	SLC2A4	TSC22D1	SDF2	NDUFB6	BC038156
SUCNR1	SUMO3	CAPNS1	MPDU1	CLTA	APOE
HEBP1	MYADM	B4GALT1	1190002H23RIK	GMPR	GRB10
LGMN	ATP5L	DAD1	CHIC2	PPP1R8	TINAGL
LY6C1	TPPP3	UBE2E3	PDK4	RAC2	BCKDK
CXCL1	EPS8	CXX1A	AHNAK	PLTP	2700078K21RIK
TIMP4	SOD3	RPS2	CXCL9	KCNJ8	CDK2AP2
LOC100045567	IDH1	CAR3	IARS2	LOC100047653	ACSF3
CEBPA	ITGA1	BMP3	WWTR1	MED11	PPAPDC1
PLA1A	CYC1	GPD1	RPS7	EG622320	SPCS1
CISH	PHGDH	NDUFS2	MTPN	FCGRT	A730008L03RIK
COL4A2	EMP1	THBS2	PODN	AP2A2	SNTG2
SERPING1	S100A8	MRAP	CYBA	PPAP2C	MTAP
TSPO	DARC	SCARA5	CHURC1	TMEM41A	ORAI3
PHLDA3	1190005I06RIK	PCBP1	RTN2	KDELR2	D930001I22RIK
RPS6	PRDX3	2310044H10RIK	PPCS	LRRC8	RPS4X
MMP2	MNAT1	OTTMUSG00000000971	OLFM1	AW146242	CCM2
PRKCB1	NDUFC2	TMEM9B	MGL2	DHRS7	CASP6
CTSF	2310008M10RIK	MRVI1	TMEM43	NFKB1	MAPK14
SLIT3	TSPAN3	NOMO1	CALU	HFE	IL2RG
H2-EB1	COL6A2	MRC1	EPB4.1L1	EPDR1	TBC1D9B
ENC1	PLIN	TGFBR2	FGFRL1	EAR2	TMEM16K
CD74	5730437N04RIK	LOC100047353	CIITA	VPS35	TPST1
EG667977	ALOX5AP	RAB18	PEPD	ITIH5	CAML
LEP	LOC100043257	SGTA	BC051227	IL1B	CTSH
EHD2	POLD4	RBCK1	L7RN6	TIMP1	ROCK1

Table S3. *Cont.*

EEF2	BC004044	SLC11A2	MGC41689	COPZ1	PPP5C
SORL1	VNN3	ERRFI1	THRA	PTGES	SCAMP3
TRP53INP2	C1QA	BC013712	MAN2A1	MGST1	HRAS1
4833421E05RIK	9030224M15RIK	TNNC2	ACAA2	MRPL34	

Table S4. Validated genes by qRT-PCR.

Upregulated Genes		Downregulated Genes
DEFB37	CLDN3	VIM
DEFB38	PDZK1	LUM
PRM1	ADAM3	MAPK1
SPINT4	SEC11C	ATP5F1
DEFB39	DEFB15	FSTL1
OAZ3	MRPL52	RARRES2
SMCP	POLR2G	CD14
NPY	YWHAZ	PRKCB1
FABP9	DEFB21	GPX7
SPINK2	COX6C	MKNK1
SPINK8	TCP11	HSD11B1
TNP1	COX8C	SOD3
CUZD1	CRISP4	PRKCB1
SPINK11	CLDN11	LOX
DEFB2	SLFNL1	TNFRSF1A
TSSK6	LRRC50	FOXO1
RPL3L	GRB7	CYBA
WFDC15B	PTGDS	TGFBI
DEFB11	ATP13A4	HRAS1
WFDC10	DEFB40	NFKB1
SPINLW1	SPINK10	MAPK1

Table S4. *Cont.*

**Upregulated Genes**		**Downregulated Genes**
LDHC	CDH16	MAPK14
PGK2	KRT18	ROCK1
SPATA19	DEFB30	PPP5C
GSG1	HRASLS5	HRAS1
ZNRF4	LTF	
SERPINA1F	EIF3K	
CLGN	CYP1B1	
KCNK1	PTPN1	
TSGA8	SPHK1	
